# Supplementary material for: Empirical methods for controlling false positives and estimating confidence in ChIP-Seq peaks
Source: BMC Bioinformatics. 2008 Dec 5;9:523. doi: 10.1186/1471-2105-9-523 (PMC2628906; doi:10.1186/1471-2105-9-523)
Supplement: Additional File 1 — A variety of html documents from the USeq web site detailing the available applications, their best usage, output file type descriptions, command line menus, etc. [file 1471-2105-9-523-S1.zip › USeqUserGuides/JavaDoc.zip!/JavaDoc/overview-summary.html]

Overview


|  |  |  |
| --- | --- | --- |
| **Overview** \| Package \| Class \| Use \| **Tree** \| **Deprecated** \| **Index** \| **Help** | |  |
| PREV   NEXT | **FRAMES**    **NO FRAMES**     **All Classes** |  |


---

| **Packages** | |
| --- | --- |
| **bioroot** |  |
| **bioroot.antibody** |  |
| **bioroot.oligo** |  |
| **bioroot.parsers** |  |
| **bioroot.plasmid** |  |
| **bioroot.strain** |  |
| **cgar** |  |
| **cgar.beans** |  |
| **edu.utah.seq.analysis** | Primary analysis applications and helper classes for working with binary PointData, xxx.bar.zip files. |
| **edu.utah.seq.compression** |  |
| **edu.utah.seq.data** | Helper classes for working with binary PointData, EnrichedRegions, etc. |
| **edu.utah.seq.methylation454** | Applications for working with 454 data and identifying bisulfite converted bases for methylation studies. |
| **edu.utah.seq.misc** |  |
| **edu.utah.seq.parsers** | Parsers for converting sequencing data into binary PointData, xxx.bar.zip files. |
| **expr** | Classes and tools related to expression array analysis. |
| **gata.aligner** | For aligning and post processing DNA sequences using BLAST for subsequent import into GATAPlotter. |
| **gata.geneGlyphs** | For representing annotation, scale bars, and generic tracks. |
| **gata.main** | Main app, utility, and params for GATAPlotter. |
| **gata.menu** | GATAPlotter menus, panels, and frames. |
| **gata.plotter** | GATAPlotter primary panel, sliders, tools, and conservation panels. |
| **igb.util** | Utilities for converting TiMAT results into Affymetrix IGB visualizations. |
| **meme** | Wrapper for meme tweaked for processing multiple rounds of SELEX oligos. |
| **selex** | For processing of raw SELEX sequence data. |
| **trans.anno** | For associating intervals, binding regions, and binding peaks with GFF annotation. |
| **trans.bkgrnd** | For examining background GC content and ratio bias. |
| **trans.cel** | Apps and methods for manipulating cel files. |
| **trans.graphics** | For graphically representing '.cel' files and intervals. |
| **trans.main** | Principle classes and scripts used in TiMAT2 analysis. |
| **trans.misc** | Rough working scripts. |
| **trans.qc** | Quality control apps for cel files. |
| **trans.roc** | For manipulating the BAC dmel spike in data and generating ROC curves. |
| **trans.tpmap** | For maping, filtering, and calculating statistics about text version bpmap files. |
| **util.amazon** |  |
| **util.apps** | Scripts for printing arrays, manipulating files, scatter plots... |
| **util.apps.gump** |  |
| **util.bio.annotation** | For representing gene annotation. |
| **util.bio.calc** | Calculators for aligments and nucleic acids. |
| **util.bio.cluster** | Clustering apps and methods. |
| **util.bio.converters** | Bio format converters. |
| **util.bio.digest** | Restriction enzyme digestion of DNA sequences. |
| **util.bio.parsers** | Parsers and extractors, fasta, quality, text, gff.... |
| **util.bio.parsers.gff** | GFF2 and GFF3 parsers. |
| **util.bio.seq** | Nucleic acid sequence manipulation. |
| **util.bio.wrappers** | Wrappers for other bio applications. |
| **util.bio.xamer** | Generation and manipulation of random sequence words of various lengths. |
| **util.gen** | Variety of helper methods for Email, IO, Encryption, HTML, Swing... |
| **util.xml** |  |

---


|  |  |  |
| --- | --- | --- |
| **Overview** \| Package \| Class \| Use \| **Tree** \| **Deprecated** \| **Index** \| **Help** | |  |
| PREV   NEXT | **FRAMES**    **NO FRAMES**     **All Classes** |  |


---
